# Single‐cell Raman and functional gene analyses reveal microbial P solubilization in agriculture waste‐modified soils

**DOI:** 10.1002/mlf2.12053

**Published:** 2023-06-14

**Authors:** Hongzhe Li, Jiazhi Ding, Longji Zhu, Fei Xu, Wenjing Li, Yanpo Yao, Li Cui

**Affiliations:** ^1^ Key Lab of Urban Environment and Health Institute of Urban Environment, Chinese Academy of Sciences Xiamen China; ^2^ College of Life Sciences Fujian Agriculture and Forestry University Fuzhou China; ^3^ University of Chinese Academy of Sciences Beijing China; ^4^ Agro‐Environmental Protection Institute Ministry of Agriculture and Rural Affairs Tianjin China

**Keywords:** CNP functional genes, D_2_O isotope labeling, phosphate‐solubilizing microorganisms, single‐cell Raman

## Abstract

Application of agricultural waste such as rapeseed meal (RM) is regarded as a sustainable way to improve soil phosphorus (P) availability by direct nutrient supply and stimulation of native phosphate‐solubilizing microorganisms (PSMs) in soils. However, exploration of the in situ microbial P solubilizing function in soils remains a challenge. Here, by applying both phenotype‐based single‐cell Raman with D_2_O labeling (Raman‐D_2_O) and genotype‐based high‐throughput chips targeting carbon, nitrogen and P (CNP) functional genes, the effect of RM application on microbial P solubilization in three typical farmland soils was investigated. The abundances of PSMs increased in two alkaline soils after RM application identified by single‐cell Raman D_2_O. RM application reduced the diversity of bacterial communities and increased the abundance of a few bacteria with reported P solubilization function. Genotypic analysis indicated that RM addition generally increased the relative abundance of CNP functional genes. A correlation analysis of the abundance of active PSMs with the abundance of soil microbes or functional genes was carried out to decipher the linkage between the phenotype and genotype of PSMs. *Myxococcota* and C degradation genes were found to potentially contribute to the enhanced microbial P release following RM application. This work provides important new insights into the in situ function of soil PSMs. It will lead to better harnessing of agricultural waste to mobilize soil legacy P and mitigate the P crisis.

## Impact statement

Stimulation of native soil microorganisms to release soil P stocks is a sustainable means of agricultural production, but faces a major challenge in terms of the deciphering of in situ microbial phosphorus (P) solubilization functions. In this study, single‐cell Raman spectroscopy and carbon, nitrogen and P (CNP) functional gene chips were used to reveal the phenotypes and genotypes of microbial P solubilization function in three farmland soils amended with agricultural waste of rapeseed meal (RM). Effects of RM on soil properties, active phosphate‐solubilizing bacteria, and CNP functional genes that may contribute to P solubilization were revealed, providing important insights into the use of agricultural waste to improve P utilization efficiency.

## INTRODUCTION

Phosphorus (P) is an essential element for agriculture production but has the most limited bioavailability in soils compared to other plant nutrients^1^. Large amounts of P fertilizers have been applied to soils, but only a small fraction (around 10%) of P is bioavailable for crop growth, with most of the P being fixed in soils in either insoluble organic or inorganic forms^2^, ^3^, ^4^. This leads to a paradoxical situation wherein the soils are rich in fixed‐P but the plants are deficient in P. Mineral P fertilizer derived from phosphate rocks is a nonrenewable resource that has been predicted to be depleted by 2100^5^. Mobilization of the soil fixed‐P for agricultural production provides a sustainable way to reduce P fertilization and mitigate the P crisis. Soil microorganisms play a crucial role in mobilizing recalcitrant soil P. Naturally occurring phosphate‐solubilizing microorganisms (PSMs) in soils can secrete phosphatases to mineralize organic P or produce organic acids to solubilize inorganic fixed‐P^6^, ^7^, ^8^. Moreover, amendments of soils with organic nutrients, such as carbon (cellulose, lignin, and glucose), organic fertilizers, and crop straw, can enhance P turnover by regulating both soil chemical (C, N, and P) and microbial properties (PSM population and enzyme activity)^9^. These organic fertilizers such as rice straw and livestock manure are presently encouraged to apply to soils for sustainable agricultural production^10^. Rapeseed meal (RM), a by‐product of rapeseed oil extract, is an environmentally friendly agriculture waste. It has richer nutrients compared to manure fertilizer and has great potential for use toward improving soil quality and driving microbial mobilization of soil‐fixed P^11^. However, the effects of RM application on soil P pool, microbial P‐solubilization activity, and the interaction among the functional genes are largely unknown.

Despite the important role of PSMs in mobilizing soil fixed‐P, it remains a major challenge to study the in situ function of PSMs in native soils because the vast majority of soil bacteria are still uncultured^12^. Presently, neither the physiology studies of PSM isolates based on culture‐dependent methods nor genomic databases on culture‐independent metagenome or PCR can reliably predict the microbial function and activity in their native habitat. Single‐cell Raman spectroscopy provides a novel culture‐independent means of in situ phenotyping bacterial function and activity based on the intrinsic biomolecules of microbial cells^13^, ^14^. When combined with stable isotope probing (SIP) such as ^13^C, ^15^N, and ^2^deuterium (D), remarkable Raman shifts induced by SIP assimilation can act as biomarkers for diverse microbial functions, such as N_2_/CO_2_ fixation, drought tolerance, and antibiotic resistance^13^, ^15^, ^16^, ^17^, ^18^, ^19^. Recently, Li et al.^18^ developed single‐cell Raman with D_2_O labeling (Raman D_2_O) as a new activity‐based approach to identify active PSMs in native soils. It is based on the finding that PSMs are more active in utilizing fixed P for their metabolism by incorporating more D for de novo synthesis than non‐PSMs. The generated C–D Raman band intensities displayed a linear relationship with microbial P solubilization ability, enabling further quantification of the P solubilization activity of individual cells. This new Raman D_2_O provides an important phenotypic approach to quantifying the abundance and in situ activity of PSMs in soils amended with RM agriculture wastes.

In addition to the phenotype characterization, it is also important to understand the underlying P solubilization mechanisms. P cycling is not independent but interacts with C and N cycling. The concentrations of soil organic C were found to regulate P cycling‐related functional genes in agroecosystems because organic C mineralization is usually accompanied by hydrolysis of organic P^20^. In addition, N input significantly increased the abundance of PSMs and the microbial phosphatase activity^21^. The recently developed quantitative microbial element cycling (QMEC) smart chip can target 71 microbial CNP functional genes, including organic P mineralization genes (e.g., *bpp*, *pccA*, and *phnK*), inorganic P solubilization genes (e.g., *pqqC*, *ppK*, and *pmoA*), C degradation genes (e.g., *amyA*, *xylA*, and *hzsB*), and N nitrification genes (e.g., *amoA* and *gdh*)^22^. As these functional genes are related to fixed P release, quantification of the abundance of CNP functional genes is a useful way to understand how soil microorganisms respond to different fertilization strategies^23^. By using the QMEC chip, the abundances of CNP functional genes have been demonstrated to significantly increase in soils with a high starter P fertilization^24^. It is anticipated that coupling the phenotypic single‐cell Raman approach with the genotypic functional gene detection will provide a comprehensive understanding of the impact of RM on microbial P solubilization in soils.

In this study, we established a microcosm experiment in which RM was added to three typical farmland soils. Both phenotypic single‐cell Raman D_2_O and genotypic CNP gene chips were applied to study the microbial P cycling in these agriculture waste‐amended soils. Together with determination of soil properties and soil microbiome sequencing, we aimed to (1) evaluate the effect of RM on soil properties, P‐solubilizing activities of native soil microbes, and soil type dependence; (2) reveal the associated soil microbiome involved in P solubilization; and (3) decipher the CNP cycling genes and interaction between C, N, and P functional genes. This study provides important insights into the use of agriculture waste to promote the release of soil‐fixed P by functional soil microbes, thereby leading to mitigation of the P crisis and a sustainable agriculture.

## RESULTS

### Effect of RM addition on soil and RM properties analysis

**Figure 1:**
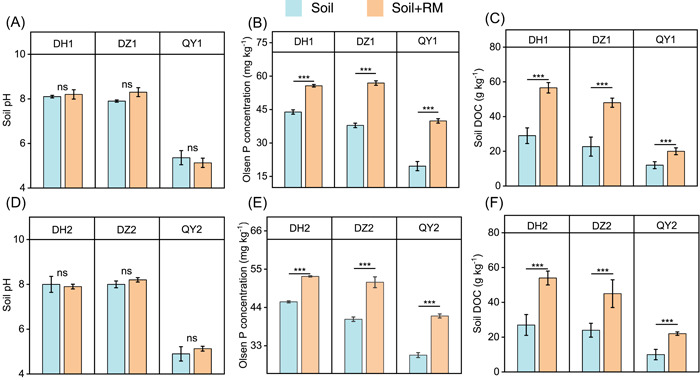
Changes in soil properties following rapeseed meal (RM) amendment. Effects of RM addition on soil pH (A, D), Olsen P concentration (B, E), and dissolved organic carbon (DOC) (C, F). Data from three replicates (*n* = 3) are represented as means ± SD. One‐way analysis of variance was used to test the significant difference between samples. DH, DZ and QY represented the soils sampled from Donghu, Dezhou and Qiyang, respectively. The numbers 1 and 2 represent the first (30 days) and the second (60 days) sampling time, respectively. ***p < 0.001; ns, no significance.

Soil samples of Dezhou (DZ) and Donghu (DH) soils were detected to be alkaline (pH > 7.5, Figure 1A,D), while Qiyang (QY) soils were acidic (pH < 6.5, Figure 1A,D). The concentrations of total P of soils and the properties of RM are shown in Tables S1 and S2. The concentrations of total P in DH soils were significantly higher than that in DZ and QY soils. A 60‐day microcosm experiment was designed to stabilize the soil microbial community after RM addition. The RM addition was found to exert no effect on soil pH in any of the treatments (*p* > 0.05, Figure 1A,D), while the concentrations of both Olsen P and dissolved organic carbon (DOC) were significantly increased in three RM‐treated soils at both sampling times of 30 and 60 days (*p* < 0.001, Figure 1B,C,E,F).

### Effect of RM addition on the phenotypic microbial P solubilization function

**Figure 2:**
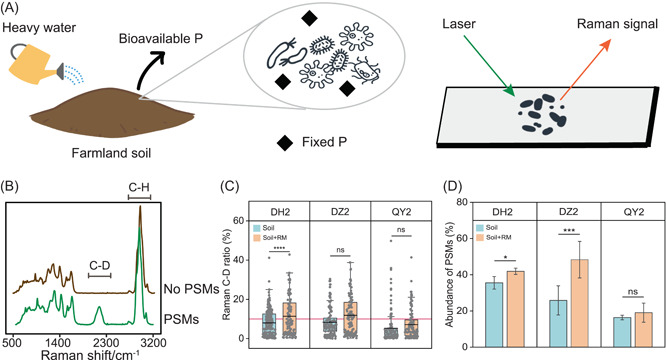
The effect of RM addition on phenotypic microbial P solubilization function. (A) Workflow for in situ phosphate‐solubilizing microorganism (PSM) identification via single‐cell Raman D_2_O. (B) Typical single‐cell Raman spectra of PSMs and non‐PSMs. (C) Distribution of C–D ratios measured from over 100 randomly selected single cells in DH, DZ, and QY soils amended with and without RM addition. Each point shows a measurement of a single cell. The red line at 10% shows the threshold for PSM identification. It was calculated as the mean + 3 × SD of C−D ratios from randomly selected bacteria incubated without D_2_O. (D) The effects of RM addition on the abundance of PSMs in DH, DZ, and QY soils identified by single‐cell Raman D_2_O. *p < 0.05, ***p < 0.001, ****p < 0.0001; ns, no significance.

After adding D_2_O to the three soils from which soluble P was removed, single‐cell Raman spectroscopy was used to study the PSMs that can actively mobilize fixed P in soils (Figure 2A). A total of over 100 single cells extracted from each soil were measured. Figure 2B shows the typical spectra of individual PSMs and non‐PSMs with distinct C–D band intensities due to their different abilities of metabolizing D in only fixed P‐containing soils. Figure 2C shows the C–D ratios of all the measured individual bacteria from the three soils at the second sampling time. Vast distributions of C–D ratios were observed, indicating the high activity heterogeneity of soil microbes in solubilizing fixed P. Additionally, RM application significantly increased the metabolic activity of PSMs in DH soils (*p* < 0.0001), but not in DZ and QY soils (*p* > 0.05).  The abundance of PSMs was calculated by dividing the number of PSMs by the total number of Raman‐detected bacteria (Figure 2D). We found that RM application significantly increased the abundance of PSMs in DH (*p* < 0.05) and DZ soils (*p* < 0.001) but not in QY soils (*p* > 0.05). These results indicated the soil‐type‐dependent effect of RM application on the phenotypic microbial P solubilization. The nonsignificant effect of RM on the QY soils may be related to its low pH, which could inhibit bacterial metabolism activity. It is noteworthy that single‐cell Raman D_2_O enabled the direct examination of individual PSMs and in situ P‐solubilization activity in native soils, which is not achievable using traditional bulk phenotypic methods such as soil enzyme activity and soil respiration rate.

### Effect of RM addition on bacterial communities

**Figure 3:**
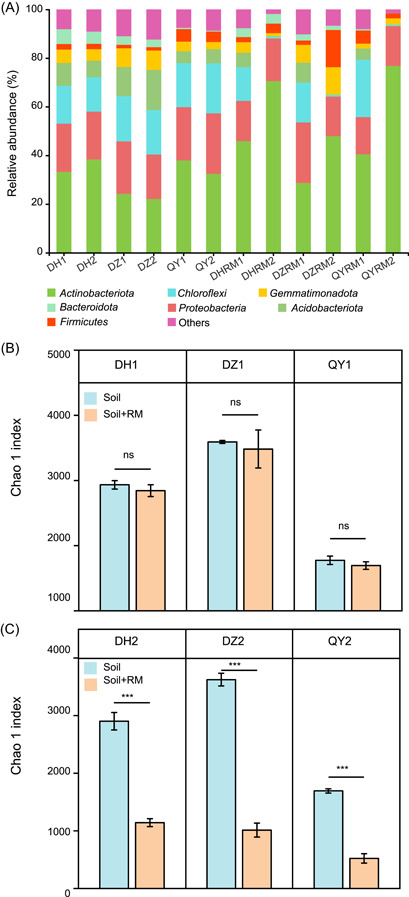
The effect of RM addition on soil bacterial community. (A) Soil bacterial community composition (mean, *n* = 3) at the phylum level. Low‐abundance taxa were classified as “Others”. The alpha diversity (Chao 1 index) of bacterial communities at the first (30 days) (B) and second (60 days) (C) sampling time. ***p < 0.001; ns, no significance.

To further understand the changes of soil microbial communities impacted by RM, a total of 1,263,169 high‐quality sequences were obtained after merging and quality filtering from 36 samples, with each sample ranging from 27,326 to 45,496. *Actinobacteriota*, *Chloroflexi*, *Gemmatimonadota*, *Bacteroidota*, *Proteobacteria*, *Acidobacteriota*, and *Firmicutes* were the seven dominant phyla in all soil samples (Figure 3A). A higher relative abundance of *Acidobacteriota* was observed in RM treatment samples compared to the control (*p* < 0.05). No significant effect of RM application on bacterial alpha diversity (Chao 1 index) was observed in soil samples at the first sampling time (Figure 3B, *p* > 0.05), while RM application significantly decreased the alpha diversity at the second sampling time in all three soils (Figure 3C, *p* < 0.001), indicating that the rich nutrient input of RM decreased microbial diversity by potentially increasing the interspecific competition and enriching some species. Principal co‐ordinates analysis (PCoA) with the Bray–Curtis distance revealed no significant difference in the bacterial communities with RM application (Figure S1, *p* > 0.05). Bacterial co‐occurrence network was further applied to analyze bacterial community associations (Figure S2). The average clustering coefficient of the network was found to increase with RM application by 0.119. More edges and labels were observed in RM treatments compared to the control (Figure S2). These results indicated that RM application enhanced the interaction of bacterial community (relative abundance > 0.1%). The sensitive taxa after RM application in three soils were analyzed by linear discriminant analysis effect size (LEfSe) (*p* < 0.05, LDA > 3.0, Figures S3–S5). The results revealed multiple bacteria as the biomarkers sensitive to RM application. For example, *genus_Acitinomadura* and *class_Myxococcia* were sensitive to RM addition in DZ, DH, and QY soils. The results indicated that RM application could enrich some bacteria in soils.

### Effect of RM addition on functional gene profiles

**Figure 4:**
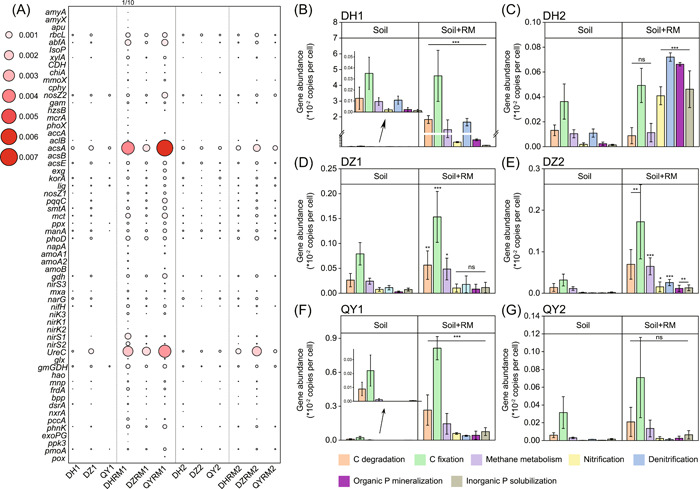
Changes of functional gene profiles following RM amendment. (A) Heat map of CNP functional gene profiles. The size of circles represents the relative abundance of CNP functional genes. The changes of the relative abundance of CNP functional gene profiles in DH (B, C), DZ (D, E), and QY (F, G) soils with and without RM application are shown. The numbers 1 and 2 represent the first (30 days) and second (60 days) sampling time, respectively. The differences were calculated as the relative abundance of CNP cycling genes between soil and soil + RM treatments. *p < 0.05, **p < 0.01, ***p < 0.001; ns, no significance.

**Figure 5:**
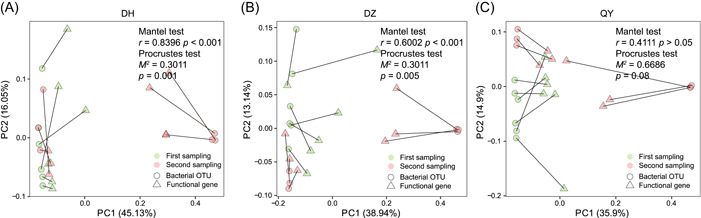
The correlation between soil bacterial communities and functional gene profiles. Procrustes analysis and Mantel test reveal the correlation between CNP functional genes and bacterial communities on the basis of Bray–Curtis dissimilarity metrics in DH (A), DZ (B), and QY (C) soils, respectively.

A total of 61 CNP cycling‐related functional genes were detected in all soil samples (Figure 4A). These CNP genes were divided into seven groups based on the main element cycling involved, that is, C cycling genes (C degradation, C fixation, and methane metabolism), N cycling genes (nitrification and denitrification), and P cycling genes (organic P mineralization and inorganic P biosynthesis). Both soil types and RM application were found to affect the relative abundance of CNP functional genes (Figure 4B–G), and RM application also changed the composition of CNP genes in all soil samples (*p* < 0.05, Figure S6). In general, the abundances of CNP functional genes in RM‐amended soils were significantly higher than those in the control (Figure 4B–G). More specifically, organic P mineralization and inorganic P solubilization genes were significantly increased following RM addition in DH and DZ2 soils (*p* < 0.01, Figure 4B,C,E), but not in QY2 soils at the second sampling time (*p* > 0.05, Figure 4G). This result is consistent with the increased abundance of phenotypic PSMs in DZ2 and DH2 soils observed by Raman D_2_O (Figure 2D). C cycling‐related genes responsible for starch, hemicellulose, and cellulose degradation were detected in all samples. With the RM addition, the relative abundance of C degradation genes increased in almost all soils (*p* < 0.01, Figure 4B, D–F), except DH and QY soils at the second sampling time, suggesting that RM addition can potentially enhance carbon‐degrading processes. The abundance of C fixation genes was much higher than C degradation genes, and significantly increased with RM addition in most soil samples (*p* < 0.01, Figure 4B,D–F). The abundance of nitrogen cycling genes also increased in most soil samples with RM addition (*p* < 0.05, Figure 4B,C,E,F). Furthermore, redundancy analysis (RDA) of the correlation between CNP functional gene profiles and environmental factors indicated that pH, DOC, and Olsen P were positively correlated with CNP functional gene profiles in all soil samples (Figure S7). Procrustes analysis and Mantel test revealed that bacterial communities were significantly correlated with CNP functional genes in the DZ and DH soils (*p* < 0.001, Figure 5A,B), but not in QY soils (*p* > 0.05, Figure 5C).

### Association of microbial P solubilization ability with soil microbiome and functional genes

**Figure 6:**
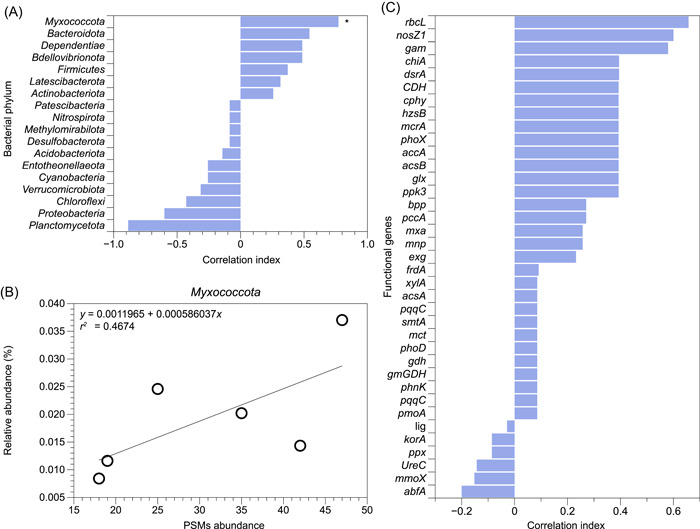
The correlations between bacterial communities, functional gene profiles, and the abundance of phosphate‐solubilizing microorganisms (PSMs). (A) Correlations between the relative abundances of bacteria at the phylum level and the proportions of active PSMs (*p < 0.05). (B) Regression relationships between the abundances of *Myxococcota* and the proportions of active PSMs. (C) Correlations between the relative abundances of functional genes and the proportions of PSMs.

The associations between phenotypes (abundance of active PSMs) and genotypes (soil microbiome and functional genes) were further analyzed. Regarding the correlations between the abundance of soil microbiome at the phylum level and the abundance of active PSMs, *Myxococcota*, *Bacteroidota*, *Dependentiae*, and *Bdellovibrionota* (correlation index > 0.5), which were previously reported to be capable of inorganic P solubilization, were positively correlated with the proportion of active PSMs (Figure 6A). Among them, *Myxococcota* was positively and significantly associated with the proportion of PSMs (*p* < 0.05, *R* ^2^ = 0.467, Figure 6B). In addition, the functional genes of *rbcL*, *nosZ1*, and *gam* (correlation index > 0.5, Figure 6C), which encode starch, pectin, and lignin hydrolysis, respectively, were positively correlated with the abundance of phenotypic PSMs.

## DISCUSSION

### Effect of RM amendments on soil properties and P‐solubilizing activities of soil microbiome

This study demonstrated that RM application not only improved soil quality by increasing soil DOC and Olsen P in all three soil types but also enhanced soil type‐dependent microbial P solubilization activity. RM is a rich nutrient source that contains high contents of P, free amino nitrogen, and carbon, explaining the improvement of soil C and P contents following RM addition^25^, ^26^, ^27^. In addition, single‐cell Raman D_2_O was applied to directly identify phenotypically active PSMs in native soils and quantify their abundance in a culture‐independent way. A clear soil type‐dependent effect of RM addition on microbial P solubilization was observed, that is, RM application significantly increased the abundance of active PSMs in two alkaline DZ and DH soils but exerted no effect on acid QY soils.

The accelerated microbial P solubilization could be related to the carbon input from RM. Previous work has found that cellulose, hemicelluloses, and lignin significantly increased the abundance of PSMs and their activity of phosphatase^28^, ^29^. For example, polysaccharide breakdown and sugar metabolism are important for stimulating phosphate solubilization^30^. In addition, organic fertilizer was found to increase the abundance of transcriptionally active *phoD*‐harboring bacteria^31^, ^32^. In our work, C degradation genes were found to increase with RM treatments, highlighting that the potential involvement of PSMs in carbon degradation may affect the metabolic traits of PSMs. In addition, considering that C mineralization usually couples with organic P hydrolysis, the addition of organic matter enhanced microbial capacity for C mineralization and P hydrolysis to maintain a balanced microbial C:P ratio in nutrient rich soils^33^. RM addition has been shown here to increase DOC in soils, thus stimulating the active PSM population. While this effects is soil type dependent. We found that RM addition increased the abundance of PSMs in alkaline DH and DZ soils but had no effect in acidic QY soils. Previous work revealed that the abundance of soil PSMs decreased with the decrease of soil pH^34^, ^35^. The reason may be that the low pH restrained the promoting effect of nutrient input on bacterial activity^36^, ^37^.

It is noteworthy that previous studies focused on microbial P solubilization function were mainly based on bulk enzyme analysis. However, this method averages out microbial P solubilization activity but could not reveal the active PSM subpopulation. In this study, Raman D_2_O achieved direct identification of active PSMs in native soils and their activity heterogeneity at the high single‐cell resolution. The PSMs with high activity of releasing fixed P and utilizing complex organic matter have great potential as bacterial fertilizers. Future studies combining single‐cell sorting and cultivation of key active PSMs will help to explore novel functional bacterial resources to alleviate the P crisis.

### Soil microbiome involved in P solubilization in three RM‐amended soils

Bacterial diversity is a fundamental factor in the soil ecosystem and plays an important role in nutrient cycling. Our study found that RM application significantly decreased the diversity of soil bacteria. The increased DOC and Olsen P following RM application change the soil substrate pool, which may stimulate the proliferation of RM utilizers but inhibit non‐RM utilizers, especially for the microorganisms that tend to live in oligotrophic habitats, thereby reducing the diversity of soil microorganisms^38^. Moreover, PCoA analysis revealed no significant difference in the bacterial community structure, suggesting that only the low‐abundance bacteria were eliminated through RM application without affecting the structure of high‐abundance bacteria. Similar effects of nutrient input on soil bacterial communities were also reported previously^39^. Co‐occurrence network analysis visualizing the scenarios of bacterial interactions showed that more nodes, edges, and average clustering coefficients were observed in RM‐treated soils. This result indicated that the application of RM enhances the interaction among soil microbiome. The more negative interactions of soil microorganisms with RM addition may enhanced bacterial interactions to promote their resistance to the change of soil substrate^40^.

The LEfSe analysis revealed some RM‐sensitive bacteria in three soils, including *Bacillus*, *Actinomadura*, and *Myxococcia*, which have been reported as keystone bacteria in agriculture ecosystems linked to the P solubilization function^41^, ^42^, ^43^. A further correlation analysis of the Raman‐based phenotypes with the taxonomy‐based genotypes revealed that the abundance of *Myxococcota* was significantly positively correlated with the proportion of active PSMs. Meanwhile, *Myxococcota* was also a sensitive biomarker for RM addition. This phylum is known to play an essential role in carbon cycling that could produce organic acid to promote P solubilization^44^. These results indicated that *Myxococcota* may be the potential active PSMs contributing to the solubilization of inorganic fixed P in soils.

### CNP cycling genes and the interaction between C, N, and P functional genes

To further explore the genotypic mechanisms of the observed microbial P solubilization in RM‐amended soils, a total of 61 CNP functional genes were detected and quantified by QMEC gene chips. Our results revealed that RM application significantly altered the structure of microbial functional genes. The abundance of CNP genes in most soils increased with RM addition, especially those involved in C degradation, C fixation, nitrification, denitrification, organic P mineralization, and inorganic P solubilization. Moreover, the abundance of P cycling‐related genes had significant positive linear correlations with the abundance of C and N cycling‐related genes (Figure S8). These results indicate that microbial P releasing processes are not independent but interact with C and N cycling^45^. The possible reason is that some functional bacteria driving C and N cycling, such as C degradation and N nitrification, can also produce organic acid, ATP, and protons to enhance P cycling^24^. A further correlation analysis indicated that C degradation genes of *rbcL*, *nosZ*, and *gam* were positively correlated with the abundance of active PSMs, indicating that the organic acid secretion during C degradation may mainly account for the microbial solubilization of fixed P. This was consistent with the increase of active PSM abundance in soils containing a higher DOC content following RM amendments. As such, the potential CNP functional genes that contribute to the enhanced microbial P mobilization upon RM addition were deciphered.

In conclusion, this work applied both single‐cell Raman D_2_O and CNP functional gene chips to reveal the phenotypes and genotypes of microbial P solubilization function in three types of farmland soils amended with agricultural waste of RM. RM addition increased both the soil C and P contents and the abundance of phenotypically active PSMs in native DZ and DH soils. Genotypic results indicated that RM application generally increased the abundance of CNP functional genes and enriched the abundance of P solubilization related bacteria. Further correlation analysis revealed that *Myxococcota*‐ and C degradation‐related genes may mainly contribute to the enhanced microbial P solubilization function. This study clearly demonstrates that RM is an agricultural friendly product that not only improves soil quality but also, more importantly, improves microbial P solubilization activity. The application of two culture‐independent methods established a potential link between the phenotype and genotype of soil‐native PSMs, which helped to explain the mechanisms about how RM addition increased the microbial P solubilization function. These findings will advance our understanding and harnessing of agricultural by‐product fertilization for the sustainable development of agriculture.

## MATERIALS AND METHODS

### Soil sample collection and incubation

Soil samples were collected from the farmlands located in DH: paddy soil, DZ: fluvo‐aquic soil, and QY: red soil. The surface layer (0–15 cm) of soils was taken for our study. The collected soils were air‐dried in the dark and the allogenic matter was removed by a 2 mm sieve. The RM was collected from a rap oil processing plant in Jiaxing, Zhejiang province. The RM was air‐dried and ground to homogenize the particle size before application into soils. The above‐mentioned three soils were amended without (control) or with 1% RM (10 g of RM per kg soil). Each treatment was performed in triplicate. The incubation experiments were performed with 60 g of soil in 100 ml glass beakers. The soil moisture was adjusted to 75% of field capacity. The soils were incubated in an illumination incubator at 25°C and sampled after 30 and 60 days of incubation. A total of 18 soil samples were obtained, and each sample was divided into two parts. One part was stored at −20°C for DNA extraction and the other part was used for determination of soil properties and Raman detection.

### Soil and RM property analyses

pH of soil and RM was measured after shaking soils in water at a soil to water ratio of 1:2.5 (w/v). Total P concentrations of soil and RM were determined by HClO_4_–H_2_SO_4_ digestion, followed by molybdenum‐blue colorimetric measurement^46^. Olsen P was extracted with a NaHCO_3_ solution and detected using the molybdenum‐blue colorimetric method^47^. Concentrations of dissolved organic carbon (DOC) were determined using a TOC analyzer (TOC‐LCPH).

### Single‐cell Raman spectroscopy to probe active PSMs in soils

To obtain available P‐free soil, the soil samples were washed three times with 0.5 mol/l NaHCO_3_ solution and ultrapure water, respectively, until there was no obvious chromogenic reaction detected by the molybdenum‐blue colorimetric method. Briefly, 1 g of soil was added to 30 ml of NaHCO_3_ solution, incubated at 25°C and 180 rpm for 8 h, and then centrifuged to remove the supernatant. The same procedure was applied to the ultrapure water^18^. The obtained soils were then air‐dried for 24 h. An aliquot of 200 μl of D_2_O was added to 1 g of soils to simulate soil moisture in nature. The soil samples were incubated at 37°C for 24 h before extraction of soil bacteria for Raman analysis.

Soil bacteria were extracted using a modified Nycodenz density gradient separation protocol^48^. Briefly, 1 g of soil was homogenized in 3 ml of phosphate‐buffered saline (PBS) with 20 μl of Tween 20. The soil slurry was vortexed for 20 min to detach soil‐associated bacteria. The vortexed slurry was then generally added to 3 ml of Nycodenz solution (Aladdin, 1.42 g/ml). To separate bacteria from soil particles, the slurry was centrifuged at 14,000 *g* for 90 min at 4°C. The middle layer containing bacteria was carefully collected into a clean 1.5 ml tube and washed with water to remove residual PBS. Two microliters of the obtained bacterial solution was loaded on an Al foil for single‐cell Raman measurements. A LabRAM Aramis confocal Raman microscope (HORIBA Jobin‐Yvon) was used for PSM identification. It is equipped with a 532 nm Nd:YAG laser (Laser Quantum), 300 g/mm grating, and a 100× objective (Olympus, NA = 0.09). For each bacterium, the spectrum in the range of 500–3200 cm^−1^ was obtained at an acquisition time of 9 s. All spectra were analyzed using LabSpec5 software (HORIBA Jobin‐Yvon) for baseline correction and peak intensity quantification. C–D ratios of CD/(CD + CH) were calculated to quantify the microbial activity for P solubilization. The C–H and C–D Raman peaks used for this calculation were at 2800–3100 and 2040–2300 cm^−1^, respectively.

### Soil DNA extraction, amplification, high‐throughput sequencing, and analysis

Soil DNA was extracted using the DNeasy PowerSoil Kit (QIAGEN). The quality and concentration of DNA were monitored using a Qubit 4 fluorometer and agarose gel electrophoresis. The bacterial 16S rRNA gene was amplified using primers of 515F‐806R according to the previously reported amplification conditions^49^. The obtained gene products were sequenced on an Illumina MiSeq PE 300 platform (Meiji Biological Medicine Co). Quantitative Insights Into Microbial Ecology (QIIME, version 1.9.1) was chosen to analyze the high‐throughput sequencing data^50^. Low‐quality reads and primer sequences were removed to obtain clean sequences. Only the sequences with over 97% of similarity were assigned to operational taxonomic units (OTUs) by using UCLUST^51^, and taxonomic assignment to OTUs was based on the Greengenes database.

### High‐throughput qPCR for quantifying CNP cycling functional genes

To reveal the effect of RM application on CNP cycling, a total of 72 primer sets targeting 71 functional genes involved in CNP cycling and 16S rRNA genes were measured using the QMEC chip by high‐throughput qPCR (HT‐qPCR)^22^. HT‐qPCR was performed on the Wafergen SmartChip Real‐time PCR system (Wafergen Inc.) and three technical replicates were set for each sample. The information on primers is shown in Table S3. SmartChip qPCR software (V 2.7.0.1) was used to analyze the HT‐qPCR data. The cutoff value of threshold cycle (*C_t_*) was set to 31 and the data with amplification efficiencies higher than 0.9–1.1 were discarded. The relative abundance of functional genes obtained by normalizing functional gene copy numbers with the 16S rRNA gene copy numbers was used in our study.

### Statistical analysis

Adonis analysis was used to analyze the similarities and differences of bacterial composition and presented by nonmetric multidimensional scaling plots (NMDS) using the Bray–Curtis dissimilarity matrix. Significant differences in soil chemical properties, the relative abundance of CNP functional genes, and the abundance of active PSMs were determined by one‐way analysis of variance. Gene co‐occurrence networks were constructed based on the Spearman's correlation matrix and visualized with Gephi V.0.9.2. Soil bacterial biomarkers (from the phylum level to the genus level) that were sensitive to RM addition were determined by LEfSe, and the threshold of the logarithmic LDA score was set as 3.0. Spearman's rank correlations between the proportion of PSMs and the abundance of bacteria at the phylum level and functional genes were calculated using the R function “cor. test.”. Origin 2021, R and DataGraph were used to plot the graphs.

## AUTHOR CONTRIBUTIONS

**Hongzhe Li**: Conceptualization (Lead); Data curation (Lead); Visualization (Lead); Writing—original draft (Lead); Writing—review & editing (Lead). **Jiazhi Ding**: Data curation (Supporting); Writing—review & editing (Supporting). **Longji Zhu**: Conceptualization (Supporting); Writing—review & editing (Supporting). **Fei Xu**: Conceptualization (Supporting); Writing—review & editing (Supporting). **Wenjing Li**: Data curation (Supporting); Visualization (Supporting). **Yanpo Yao**: Writing—review & editing (Supporting). **Li Cui**: Conceptualization (Lead); Funding acquisition (Lead); Writing—original draft (Lead).

## ETHICS STATEMENT

This article does not contain any studies with human participants or animals performed by any of the authors.

## CONFLICT OF INTERESTS

The authors declare no conflict of interests.

## Supporting information

Supporting information.

## Data Availability

All relevant data are available in the Supporting Information.

## References

[1] Sharma SB , Sayyed RZ , Trivedi MH , Gobi TA . Phosphate solubilizing microbes: sustainable approach for managing phosphorus deficiency in agricultural soils. SpringerPlus. 2013;2:587. 25674415 10.1186/2193-1801-2-587 PMC4320215

[2] Yang X , Post WM , Thornton PE , Jain A . The distribution of soil phosphorus for global biogeochemical modeling. Biogeosciences. 2013;10:2525–37.

[3] Sarkhot DV , Ghezzehei TA , Berhe AA . Effectiveness of biochar for sorption of ammonium and phosphate from dairy effluent. J Environ Qual. 2013;42:1545–54. 24216432 10.2134/jeq2012.0482

[4] Zhu J , Li M , Whelan M . Phosphorus activators contribute to legacy phosphorus availability in agricultural soils: a review. Sci Total Environ. 2018;612:522–37. 28865270 10.1016/j.scitotenv.2017.08.095

[5] Peñuelas J , Poulter B , Sardans J , Ciais P , Van Der Velde M , Bopp L , et al. Human‐induced nitrogen–phosphorus imbalances alter natural and managed ecosystems across the globe. Nat Commun. 2013;4:2934. 24343268 10.1038/ncomms3934

[6] Nannipieri P , Giagnoni L , Landi L , Renella G . Role of phosphatase enzymes in soil. Berlin, Heidelberg: Springer; 2011.

[7] Stout LM , Joshi SR , Kana TM , Jaisi DP . Microbial activities and phosphorus cycling: an application of oxygen isotope ratios in phosphate. Geochim Cosmochim Acta. 2014;138:101–16.

[8] Zhang L , Feng G , Declerck S . Signal beyond nutrient, fructose, exuded by an arbuscular mycorrhizal fungus triggers phytate mineralization by a phosphate solubilizing bacterium. ISME J. 2018;12:2339–51. 29899507 10.1038/s41396-018-0171-4 PMC6155042

[9] Velázquez E , Rodriguez‐Barrueco C . First international meeting on microbial phosphate solubilization. Salamanca, Spain: Springer Science & Business Media; 2007.

[10] Reganold JP , Wachter JM . Organic agriculture in the twenty‐first century. Nat Plants. 2016;2:15221. 27249193 10.1038/nplants.2015.221

[11] Moore A . Fertilizer potential of biofuel byproducts. In: Biofuel production‐recent developments and prospects. Rijeka, Croatia; 2011. p. 437–50.

[12] Hofer U . The majority is uncultured. Nat Rev Microbiol. 2018;16:716–7. 10.1038/s41579-018-0097-x 30275521

[13] Berry D , Mader E , Lee TK , Woebken D , Wang Y , Zhu D , et al. Tracking heavy water (D_2_O) incorporation for identifying and sorting active microbial cells. Proc Natl Acad Sci USA. 2015;112:E194–203. 25550518 10.1073/pnas.1420406112 PMC4299247

[14] Lorenz B , Wichmann C , Stöckel S , Rösch P , Popp J . Cultivation‐free Raman spectroscopic investigations of bacteria. TIM. 2017;25:413–24. 10.1016/j.tim.2017.01.002 28188076

[15] Jing X , Gou H , Gong Y , Su X , Xu L , Ji Y , et al. Raman‐activated cell sorting and metagenomic sequencing revealing carbon‐fixing bacteria in the ocean. Environ Microbiol. 2018;20:2241–55. 29727057 10.1111/1462-2920.14268 PMC6849569

[16] Cui L , Yang K , Li H‐Z , Zhang H , Su J‐Q , Paraskevaidi M , et al. Functional single‐cell approach to probing nitrogen‐fixing bacteria in soil communities by resonance Raman spectroscopy with 15N2 labeling. Anal Chem. 2018;90:5082–9. 29557648 10.1021/acs.analchem.7b05080

[17] Yang K , Li H‐Z , Zhu X , Su J‐Q , Ren B , Zhu Y‐G , et al. Rapid antibiotic susceptibility testing of pathogenic bacteria using heavy‐water‐labeled single‐cell Raman spectroscopy in clinical samples. Anal Chem. 2019;91:6296–303. 30942570 10.1021/acs.analchem.9b01064

[18] Li HZ , Bi Q , Yang K , Zheng B‐X , Pu Q , Cui L . D_2_O‐isotope‐labeling approach to probing phosphate‐solubilizing bacteria in complex soil communities by single‐cell Raman spectroscopy. Anal Chem. 2019;91:2239–46. 30608659 10.1021/acs.analchem.8b04820

[19] No JH , Nishu SD , Hong J‐K , Lyou ES , Kim MS , Wee GN , et al. Raman‐deuterium isotope probing and metagenomics reveal the drought tolerance of the soil microbiome and its promotion of plant growth. mSystems. 2022;7:e01249–21. 35103487 10.1128/msystems.01249-21 PMC8805637

[20] Smeck NE . Phosphorus dynamics in soils and landscapes. Geoderma. 1985;36:185–99.

[21] Widdig M , Schleuss P‐M , Weig AR , Guhr A , Biederman LA , Borer ET , et al. Nitrogen and phosphorus additions alter the abundance of phosphorus‐solubilizing bacteria and phosphatase activity in grassland soils. Front Environ Sci. 2019;7:185.

[22] Zheng B , Zhu Y , Sardans J , Peñuelas J , Su J . QMEC: a tool for high‐throughput quantitative assessment of microbial functional potential in C, N, P, and S biogeochemical cycling. Sci China Life Sci. 2018;61:1451–62. 30136056 10.1007/s11427-018-9364-7

[23] Chen Q‐L , Ding J , Zhu D , Hu H‐W , Delgado‐Baquerizo M , Ma Y‐B , et al. Rare microbial taxa as the major drivers of ecosystem multifunctionality in long‐term fertilized soils. Soil Biol Biochem. 2020;141:107686.

[24] Li H , Bi Q , Yang K , Lasson S , Zheng B , Cui L , et al. High starter phosphorus fertilization facilitates soil phosphorus turnover by promoting microbial functional interaction in an arable soil. J Environ Sci. 2020;94:179–85. 10.1016/j.jes.2020.03.040 32563482

[25] Boström D , Eriksson G , Boman C , Öhman M . Ash transformations in fluidized‐bed combustion of rapeseed meal. Energy Fuels. 2009;23:2700–6.

[26] Salakkam A , Webb C . Production of poly (3‐hydroxybutyrate) from a complete feedstock derived from biodiesel by‐products (crude glycerol and rapeseed meal). Biochem Eng J. 2018;137:358–64.

[27] Wongsirichot P , Gonzalez‐Miquel M , Winterburn J . Recent advances in rapeseed meal as alternative feedstock for industrial biotechnology. Biochem Eng J. 2022;180:108373.

[28] Nahas E . Phosphate solubilizing microorganisms: effect of carbon, nitrogen, and phosphorus sources. Springer; 2007. p. 111–5.

[29] Wu X , Cui Z , Peng J , Zhang F , Liesack W . Genome‐resolved metagenomics identifies the particular genetic traits of phosphate‐solubilizing bacteria in agricultural soil. ISME Commun. 2022;2:17. 37938650 10.1038/s43705-022-00100-z PMC9723772

[30] Li J , Lu J , Wang H , Fang Z , Wang X , Feng S , et al. A comprehensive synthesis unveils the mysteries of phosphate‐solubilizing microbes. Biol Rev. 2021;96:2771–93. 34288351 10.1111/brv.12779 PMC9291587

[31] Hu Y , Xia Y , Sun Q , Liu K , Chen X , Ge T , et al. Effects of long‐term fertilization on phoD‐harboring bacterial community in Karst soils. Sci Total Environ. 2018;628–629:53–63. 10.1016/j.scitotenv.2018.01.314 29428860

[32] Liu W , Ling N , Luo G , Guo J , Zhu C , Xu Q , et al. Active phoD‐harboring bacteria are enriched by long‐term organic fertilization. Soil Biol Biochem. 2021;152:108071.

[33] Heuck C , Weig A , Spohn M . Soil microbial biomass C: N: P stoichiometry and microbial use of organic phosphorus. Soil Biol Biochem. 2015;85:119–29.

[34] Appunu C , Dhar B . Symbiotic effectiveness of acid‐tolerant Bradyrhizobium strains with soybean in low pH soil. Afr. J Biomed Res. 2006;5:842–5.

[35] Lin Y , Ye G , Liu D , Ledgard S , Luo J , Fan J , et al. Long‐term application of lime or pig manure rather than plant residues suppressed diazotroph abundance and diversity and altered community structure in an acidic Ultisol. Soil Biol Biochem. 2018;123:218–28.

[36] Luo G , Sun B , Li L , Li M , Liu M , Zhu Y , et al. Understanding how long‐term organic amendments increase soil phosphatase activities: insight into phoD‐and phoC‐harboring functional microbial populations. Soil Biol Biochem. 2019;139:107632.

[37] Chen X , Jiang N , Condron LM , Dunfield KE , Chen Z , Wang J , et al. Soil alkaline phosphatase activity and bacterial phoD gene abundance and diversity under long‐term nitrogen and manure inputs. Geoderma. 2019;349:36–44.

[38] Siles JA , Cajthaml T , Frouz J , Margesin R . Assessment of soil microbial communities involved in cellulose utilization at two contrasting Alpine forest sites. Soil Biol Biochem. 2019;129:13–6.

[39] Tang X , Zou L , Su S , Lu Y , Zhai W , Manzoor M , et al. Long‐term manure application changes bacterial communities in rice rhizosphere and arsenic speciation in rice grains. Environ Sci Technol. 2021;55:1555–65. 33449628 10.1021/acs.est.0c03924

[40] Shi S , Nuccio EE , Shi ZJ , He Z , Zhou J , Firestone MK . The interconnected rhizosphere: high network complexity dominates rhizosphere assemblages. Ecol Lett. 2016;19:926–36. 27264635 10.1111/ele.12630

[41] Mažylytė R , Kaziūnienė J , Orola L , Valkovska V , Lastauskienė E , Gegeckas A . Phosphate solubilizing microorganism *Bacillus* sp. MVY‐004 and its significance for biomineral fertilizers' development in agrobiotechnology. Biology. 2022;11:254. 35205120 10.3390/biology11020254 PMC8869773

[42] Grady EN , MacDonald J , Liu L , Richman A , Yuan Z‐C . Current knowledge and perspectives of *Paenibacillus*: a review. Microb Cell Fact. 2016;15:203. 27905924 10.1186/s12934-016-0603-7 PMC5134293

[43] Borah A , Thakur D . Phylogenetic and functional characterization of culturable endophytic actinobacteria associated with *Camellia* spp. for growth promotion in commercial tea cultivars. Front Microbiol. 2020;11:318. 32180767 10.3389/fmicb.2020.00318 PMC7059647

[44] Langwig MV , De Anda V , Dombrowski N , Seitz KW , Rambo IM , Greening C , et al. Large‐scale protein level comparison of Deltaproteobacteria reveals cohesive metabolic groups. ISME J. 2022;16:307–20. 34331018 10.1038/s41396-021-01057-y PMC8692467

[45] Gruber N , Galloway JN . An earth‐system perspective of the global nitrogen cycle. Nature. 2008;451:293–6. 18202647 10.1038/nature06592

[46] Zhang S , Huffman T , Zhang X , Liu W , Liu Z . Spatial distribution of soil nutrient at depth in black soil of Northeast China: a case study of soil available phosphorus and total phosphorus. J Soils Sedim. 2014;14:1775–89.

[47] Mitran T , Mani PK , Basak N , Mazumder D , Roy M . Long‐term manuring and fertilization influence soil inorganic phosphorus transformation vis‐a‐vis rice yield in a rice–wheat cropping system. Arch Agron Soil Sci. 2016;62:1–18.

[48] Eichorst SA , Strasser F , Woyke T , Schintlmeister A , Wagner M , Woebken D . Advancements in the application of NanoSIMS and Raman microspectroscopy to investigate the activity of microbial cells in soils. FEMS Microbiol Ecol. 2015;91:fiv106. 26324854 10.1093/femsec/fiv106 PMC4629873

[49] Li H‐Z , Zhu D , Lindhardt JH , Lin S‐M , Ke X , Cui L . Long‐term fertilization history alters effects of microplastics on soil properties, microbial communities, and functions in diverse farmland ecosystem. Environ Sci Technol. 2021;55:4658–68. 33754703 10.1021/acs.est.0c04849

[50] Caporaso JG , Kuczynski J , Stombaugh J , Bittinger K , Bushman FD , Costello EK , et al. QIIME allows analysis of high‐throughput community sequencing data. Nat Methods. 2010;7:335–6. 20383131 10.1038/nmeth.f.303 PMC3156573

[51] Edgar RC . Search and clustering orders of magnitude faster than BLAST. Bioinformatics. 2010;26:2460–1. 20709691 10.1093/bioinformatics/btq461

