# Supplementary material for: Single‐cell Raman and functional gene analyses reveal microbial P solubilization in agriculture waste‐modified soils
Source: mLife. 2023 Jun 14;2(2):190–200. doi: 10.1002/mlf2.12053 (PMC10989763; doi:10.1002/mlf2.12053)
Supplement: Supplementary file 1 — Supporting information. [file MLF2-2-190-s001.docx]

**Supporting information**

# Single-cell Raman and functional gene analyses reveal microbial P solubilization in agriculture waste-modified soils

Hong-Zhe Li^1^, Jia-Zhi Ding^1,2,3^, Long-Ji Zhu^1^, Fei Xu^1,2,3^, Wen-Jing Li^1,3^, Yan-Po Yao^4^ and Li Cui^1*^

^1^Key Lab of Urban Environment and Health, Institute of Urban Environment, Chinese Academy of Sciences, 1799 Jimei Road, Xiamen 361021, China;

^2^College of Life Sciences, Fujian Agriculture and Forestry University, Fuzhou 350002, China

^3^University of Chinese Academy of Sciences, 19A Yuquan Road, Beijing 100049, China;

^4^Agro-Environmental Protection Institute, Ministry of Agriculture and Rural Affairs, Tianjin 300193, China

Author for corresponding:

*Li Cui*

Email: [*lcui@iue.ac.cn*](mailto:lcui@iue.ac.cn)

**Contents**

**Tables**

Table S1. The concentrations of total P in three soils.

Table S2. The properties of RM.

Table S3. The information of CNP functional gene primers.

**Figure**

Figure S1. Principal co-ordinates analysis of soil bacterial communities based on the Bray-Curtis distance. Signiﬁcant eﬀects between diﬀerent treatments were calculated using Adonis analysis. The number 1, 2 represent the ﬁrst (30 days) and second (60 days) sampling times respectively.

Figure S2. Network analysis revealing the soil bacterial co-occurrence pattern at the genus level from all samples without (A) and with RM addition (B). Positive correlations are in green and negative correlations are in red.

Figure S3. LEfSe result presenting bacterial biomarkers (from phylum level to genus level) that were sensitive to RM addition in DH soils.

Figure S4. LEfSe result presenting bacterial biomarkers (from phylum level to genus level) that were sensitive to RM addition in DZ soils.

Figure S5. LEfSe result presenting bacterial biomarkers (from phylum level to genus level) that were sensitive to RM addition in QY soils.

Figure S6. Principal co-ordinates analysis of soil CNP functional gene profiles based on the Bray-Curtis distance.

Figure S7. The contribution of environmental factors (DOC, Olsen P and pH) to the shift of CNP functional gene profiles in soils based on redundancy analysis (RDA).

Figure S8. Regression relationships between the abundance of P cycling genes and C cycling genes (A) and N cycling genes (B).

Table S1. The concentrations of total P in three soils.

| Soil type | Concentrations of total P (mg•kg^-1^) |
| --- | --- |
| DH | 215 ± 5.2 |
| DZ | 180 ± 4.2 |
| QY | 190 ± 8.0 |

Table S2. The properties of RM.

| RM property |  |
| --- | --- |
| pH | 5.8 ± 0.1 |
| Total P (mg•kg^-1^) | 1330 ± 8.7 |
| Olsen P (mg•kg^-1^) | 121.3 ± 5.7 |
| DOC (mg•kg^-1^) | 32.2 ± 1.2 |

Table S3. The information of CNP functional gene primers.

| **Target gene** | **Function** | **Forward Primers** | **Sequences** | **Reverse Primers** | **Sequences** |
| --- | --- | --- | --- | --- | --- |
| hzsB | Anaerobic ammonium oxidation | HSBeta396F | ARGGHTGGGGHAGYTGGAAG | HSBeta742R | GTYCCHACRTCATGVGTCTG |
| accA | C fixation | accA-F | GAAGGCTAYCGCAARGC | accA-R | CCTTCMGGSGARATMAC |
| aclB | C fixation | 892F | TGGACMATGGTDGCYGGKGGT | 1204R | ATAGTTKGGSCCACCTCTTC |
| acsA | C fixation | acsA-F | GATACCTGGTGGCAGACCGA | acsA-R | TGATCACGTCGTCGACCCGG |
| acsE | C fixation | acsE-F | TCATCGGCGAACGCATCAAC | acsE-R | AGRCCGGCTTCSATGGC |
| exg | C fixation | exg-F | YSTACGGSATGCACTGGMT | exg-R | TANCGCAGRTAGTCVCCCAT |
| frdA | C fixation | frdA-F | MTGCTGCACACSCTGTW | frdA-R | CCGGTSGGGTGRWACTG |
| gam | C fixation | gam-F | CGSAACTGGGAYTACCGS | gam-R | TCCCACAGSCCSKCGTC |
| gdh | C fixation | GDH-F | GCCATCGGYCCWTACAAGGG | GDH-R | ATGTCRCCNGCCGGAACGTC |
| gmGDH | C fixation | OGSH-F | ATCGCGTTCGGGCCGGACG | OGSH-R | ATSAGRTTSAGCTCGTCCCA |
| Isop | C fixation | Isop-F | GTCATYTACTTYGGNCC | Isop-R | CGNGCSACATCNGCCCA |
| korA | C fixation | korA3-F | GCCGGCTACCCCATCACCCC | korA3-R | ATGATGGGATGGTCGCCATG |
| mct | C fixation | mct2-F | TGGGCGCSGASGTSATMCG | mct2-R | TTGACSGTRTARTCSAYSGC |
| pccA | C fixation | pccA-F | GTGMTGATCAAGGCCWC | pccA-R | CGSGTGTTCATYTCSAGGAA |
| rbcL | C fixation | rbcL-F | AAGGACGACGAGAACATC | rbcL-R | TGCAGGATCATGTCGTT |
| smtA | C fixation | smtA2-F | TTTCTGGCCGGBTAYGCDGC | smtA2-R | CGGTACGGHCCGGTYTGVCC |
| CDH | Cellulose hydrolysis | cdh-F | ATWRYCTWCCGMRTHGCCMT | cdh-R | GTKAGSGGRTTBYKGRYCAT |
| exoPG | Chitin hydrolysis | exoPG-F | GATTGGTSVCAATATGAYRG | exoPG-R | STCCARCCACCRAYRCTRAA |
| mnp | Chitin hydrolysis | mnp-F | MACRCCSTTCGACTCSACC | mnp-R | ACGTCSGAGCAGTCRAYGA |
| narG | Denitrification | narG1960m2F | TAYGTSGGGCAGGARAAACTG | narG2050m2R | CGTAGAAGAAGCTGGTGCTGT |
| nirK1 | Denitrification | nirK 1F | GGMATGGTKCCSTGGCA | nirK 5R | GCCTCGATCAGRTTRTGGTT |
| nirK2 | Denitrification | nirKC1F | ATGGCGCCATCatggtnytncc | nirKC1R | TCGAAGGCCTCGatnarrttrtg |
| nirS1 | Denitrification | nirS cd3AF | GTSAACGTSAAGGARACSGG | nirS R3cd | GASTTCGGRTGSGTCTTGA |
| nirS2 | Denitrification | nirSC1F | ATCGTCAACGTCaargaracvgg | nirSC1R | TTCGGGTGCGTCttsabgaasag |
| nirS3 | Denitrification | nirSC2F | TGGAGAACGCCggncargtntgg | nirSC2R | GATGATGTCCACGgcnacrtangg |
| nosZ1 | Denitrification | nosZ-F | CGYTGTTCMTCGACAGCCAG | nosZ-R | CGSACCTTSTTGCCSTYGCG |
| nosZ2 | Denitrification | nosZ3F | CGCRACGGCAASAAGGTSMSSGT | nosZ3R | CAKRTGCAKSGCRTGGCAGAA |
| abfA | Hemicellulose hydrolysis | abfA-F | CGSTAYCCSGGCGGCAAYTT | abfA-R | TGCCASGGNCCGTCCATYTC |
| manA | Hemicellulose hydrolysis | manA-F | ATGCGCGGBGTCAACCA | manA-R | TCGTTGSCGATGTTGABGA |
| xylA | Hemicellulose hydrolysis | xylA-F | TGGGGBGGTCGYGAAGG | xylA-R | ACTTTGGCRTCRAAGTT |
| ppx | Inorganic P solubilization | ppx2-F | TGCATCTGGCGGACGGCCT | ppx2-R | AGATCCGCCGCCAATATCA |
| pqqC | Inorganic P solubilization | pqqC2-F | AACCGCTTCTACTACCAG | pqqC2-R | GCGAACAGCTCGGTCAG |
| chiA | Lignin hydrolysis | chiA-F | TSAAGAARTACGCSGACAACG | chiA-R | ASGTCATCAGRCCCTTSAG |
| glx | Lignin hydrolysis | glx-F | AACCAGTCGATCATCTACGA | glx-R | RTGSACGAGCTCDGGCATGG |
| lig | Lignin hydrolysis | Lig1-F | CCGCACACACTGTTGCTGC | Lig1-R | CGAAGGATTGCCACTCGCA |
| nifH | N fixation | nifHF | AAAGGYGGWATCGGYAARTCCACCAC | nifHRb | TGSGCYTTGTCYTCRCGGATBGGCAT |
| amoA1 | Nitrification | Arch_amoAF | STAATGGTCTGGCTTAGACG | Arch_amoAR | GCGGCCATCCATCTGTATGT |
| amoA2 | Nitrification | amoA1F | GGGGTTTCTACTGGTGGT | amoA2R | CCCCTCKGSAAAGCCTTCTT |
| amoB | Nitrification | amoBMf | TGGTAYGACATKAWATGG | amoBMr | RCGSGGCARGAACATSGG |
| nxrA | Nitrification | F1370 F1 nxrA | CAGACCGACGTGTGCGAAAG | F2843 R2 nxrA | TCCACAAGGAACGGAAGGTC |
| bpp | Organic P mineralization | BPP-F | GACGCAGCCGAYGAYCCNGCNITNTGG | BPP-R | CAGGSCGCANRTCIACRTTRTT |
| phnK | Organic P mineralization | PhnK-F | CATCGTCGGCGAATCCGG | PhnK-R | TGCTGCATGCCGCCGGAAAA |
| phoD | Organic P mineralization | ALPS-F730 | CAGTGGGACGACCACGAGGT | ALPS-R1101 | GAGGCCGATCGGCATGTCG |
| amyA | Starch hydrolysis | amyA-F | YGGTTTTCGTCTTGACGCSG | amyA-R | MGGCTGMGTRTCATGRTTK |
| apu | Starch hydrolysis | apu2-F | ACVTGGATAGGYGAGCCYCA | apu2-R | CCRTCSGGGAAGTAGTTKCC |

Figure S1. Principal co-ordinates analysis of soil bacterial communities based on the Bray-Curtis distance. Signiﬁcant eﬀects between diﬀerent treatments were calculated using Adonis analysis. The number 1, 2 represent the ﬁrst (30 days) and second (60 days) sampling times respectively.

Figure S2. Network analysis revealing the soil bacterial co-occurrence pattern at the genus level from all samples without (A) and with RM addition (B). Positive correlations are in green and negative correlations are in red.


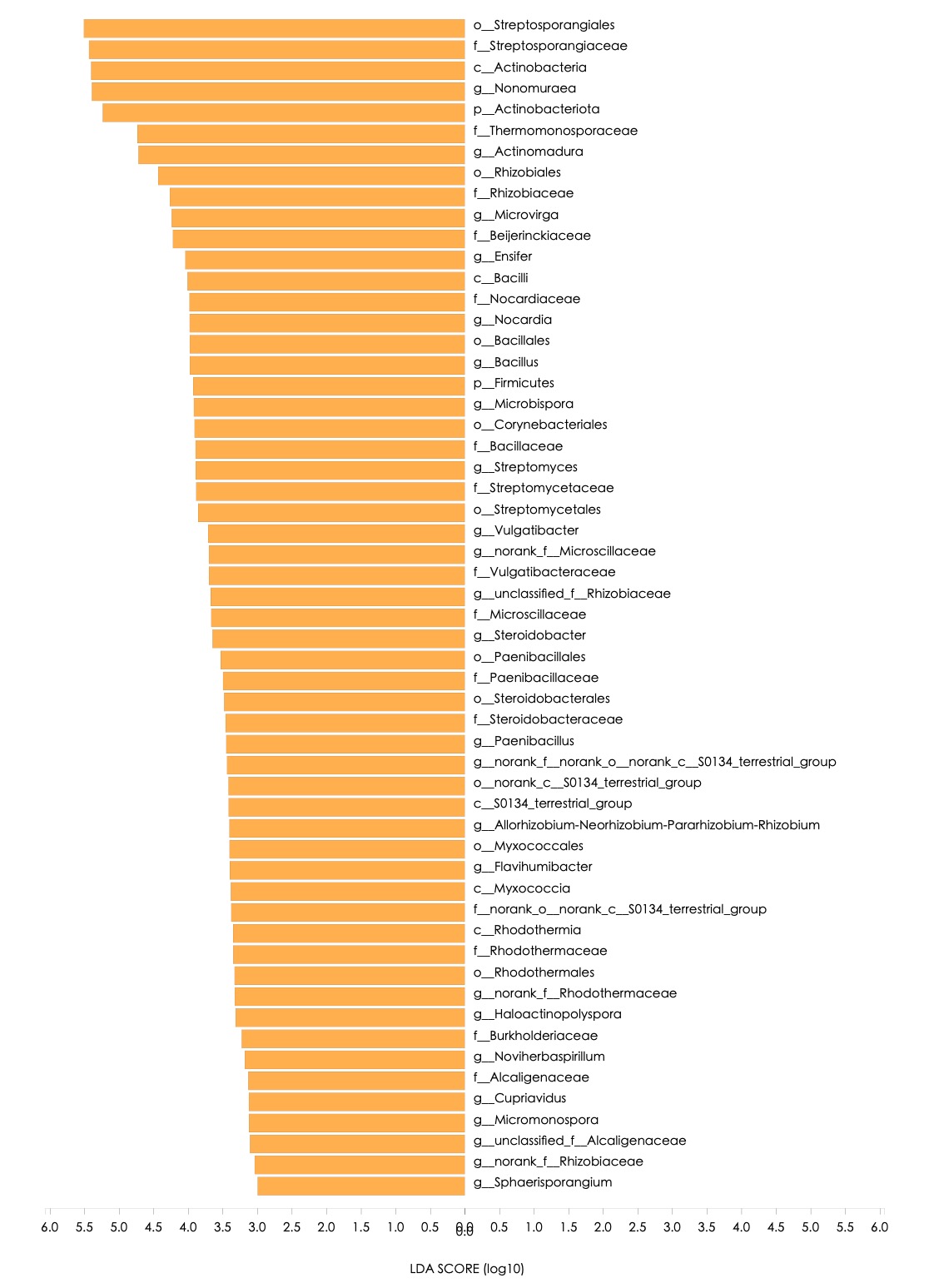


Figure S3. LEfSe result presenting bacterial biomarkers (from phylum level to genus level) that were sensitive to RM addition in DH soils.


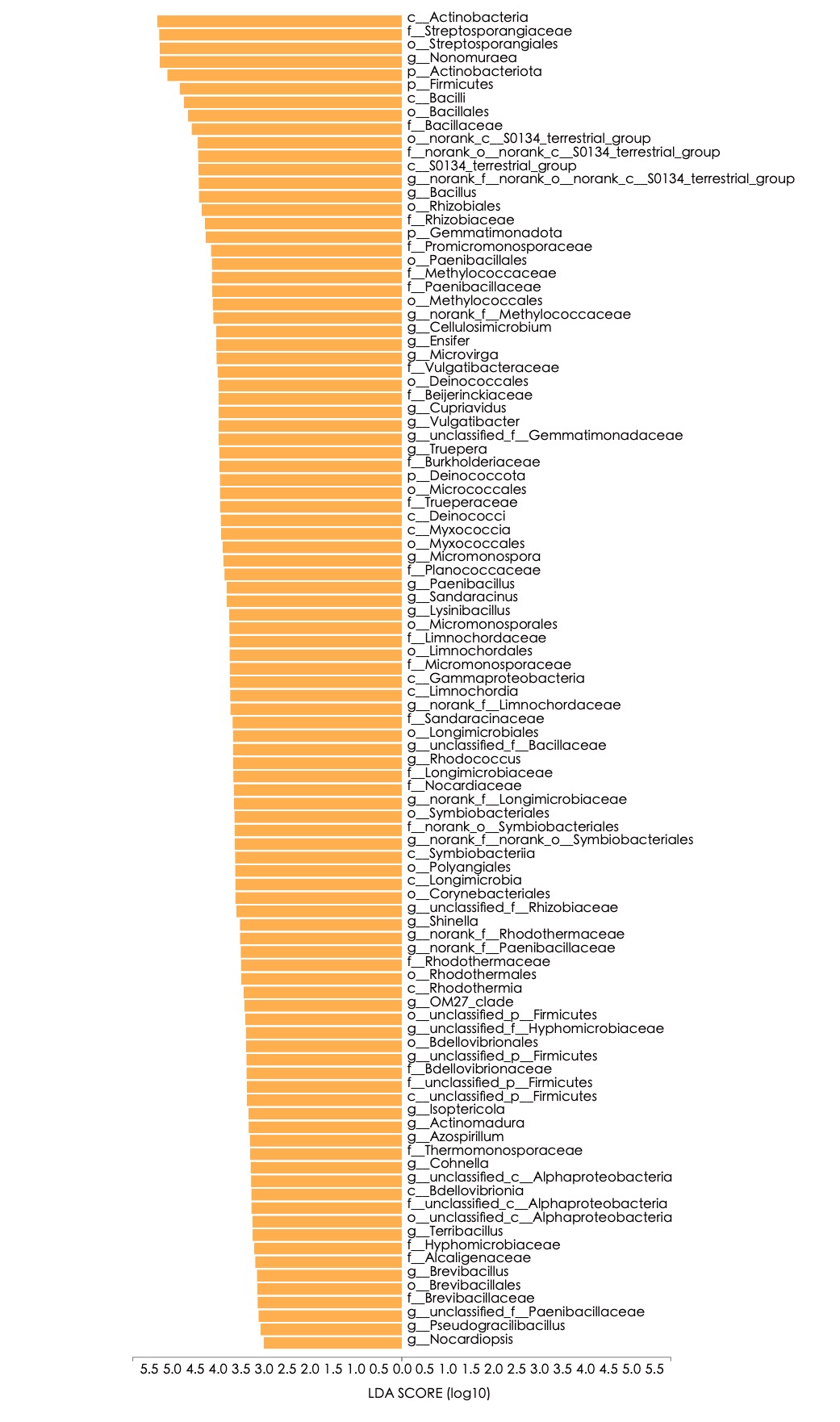


Figure S4. LEfSe result presenting bacterial biomarkers (from phylum level to genus level) that were sensitive to RM addition in DZ soils.


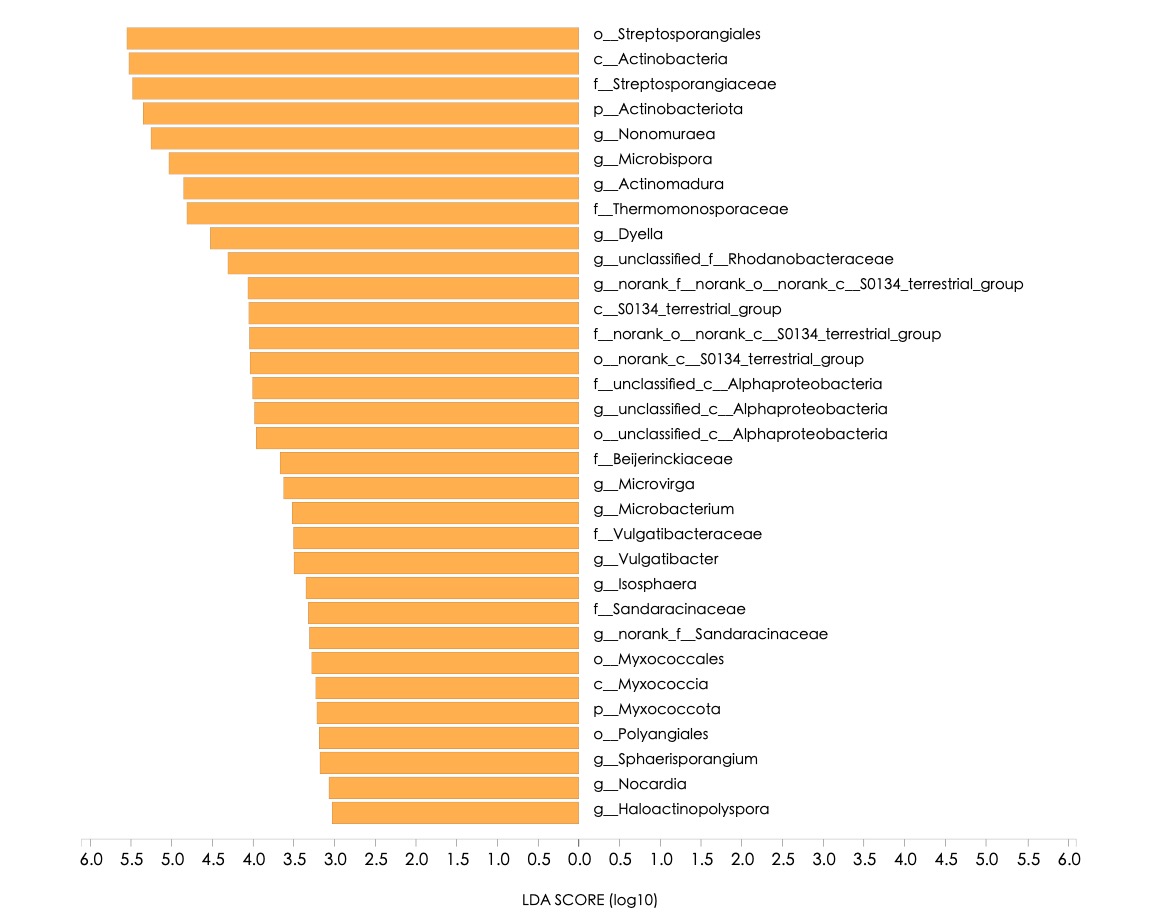


Figure S5. LEfSe result presenting bacterial biomarkers (from phylum level to genus level) that were sensitive to RM addition in QY soils.

Figure S6. Principal co-ordinates analysis of soil CNP functional gene profiles based on the Bray-Curtis distance.

Figure S7. The contribution of environmental factors (DOC, Olsen P and pH) to the shift of CNP functional gene profiles in soils based on redundancy analysis (RDA).

Figure S8. Regression relationships between the abundance of P cycling genes and C cycling genes (A) and N cycling genes (B).
